# Postoperative Adiponectin Levels in Pediatric Patients Undergoing Open Heart Surgery

**DOI:** 10.1155/2013/408680

**Published:** 2013-10-09

**Authors:** A. Thaler, H. Kanety, T. Avni, D. Mishali, R. Hemi, E. Yissaschar, C. Pariente, G. Paret, D. Modan-Moses

**Affiliations:** ^1^Pediatric Intensive Care Department, Chaim Sheba Medical Center, Safra Children's Hospital, 52621 Tel-Hashomer, Israel; ^2^Sackler School of Medicine, Tel-Aviv University, 39040 Tel-Aviv, Israel; ^3^Institute of Endocrinology, Chaim Sheba Medical Center, Safra Children's Hospital, 52621 Tel-Hashomer, Israel; ^4^Department of Cardiothoracic Surgery, Chaim Sheba Medical Center, Safra Children's Hospital, 52621 Tel-Hashomer, Israel; ^5^Pediatric Endocrinology and Diabetes Unit, Chaim Sheba Medical Center, The Edmond and Lily Safra Children's Hospital, 52621 Tel-Hashomer, Israel

## Abstract

*Background*. Adipose tissue is an important endocrine organ that secretes cytokines, including adiponectin, levels of which are negatively correlated with the severity of the inflammatory process. *Aim*. To assess the time course of adiponectin levels following open heart surgery with cardiopulmonary bypass and its correlation with early postoperative outcomes. *Materials and Methods*. Blood samples were obtained from 24 children undergoing cardiac surgery and analyzed for adiponectin, C-reactive protein, and other inflammatory markers. *Results*. Baseline adiponectin levels were negatively correlated with patients' preoperative weight and age. Postoperative adiponectin levels decreased compared to baseline (*P* = 0.01) and correlated negatively with duration of cardiopulmonary bypass (*r* = −0.438, *P* = 0.037), length of stay in the pediatric intensive care unit (*r* = −0.457, *P* = 0.025), and the inotropic score (*r* = −0.471, *P* = 0.02). Adiponectin levels were positively correlated with sVCAM 1 levels; however, there was no correlation between adiponectin levels and sP selectin, tPA, MCP1, and sCD40. *Conclusions*. The inflammatory response after open heart surgery with cardiopulmonary bypass is associated with a reduction in adiponectin levels. Prolonged or more complicated surgery induced a more substantial inflammatory process characterized by a significant reduction in adiponectin levels over time and a delayed return to baseline levels.

## 1. Introduction

Open heart surgery (OHS) involving cardiopulmonary bypass (CPB) triggers a well-recognized systemic inflammatory response. This response is thought to be due to endothelial cell injury, neutrophil activation, increased synthesis of multiple cytokines, upregulation of adhesion molecules, initiation of the coagulation cascade, and free radical formation, possibly leading to severe postoperative complications [1]. 

Adipose tissue has been demonstrated to be an important endocrine organ which secretes cytokines and regulatory factors. Adiponectin, the most abundant adipose-derived protein in humans, has significant anti-inflammatory and insulin-sensitizing effects. In contrast to other adipocytokines, adiponectin levels are inversely correlated with body fat mass [2]. Adiponectin levels are elevated at birth, increase from birth to the first month of life, and decrease over time until adulthood [3]. Adiponectin circulates in three distinct multimers: trimers (low molecular weight, LMW), hexamers (middle molecular weight, MMW), and larger multimers of 12 to 18 subunits (high molecular weight, HMM). The amount and distribution of these molecular forms can determine the activity of adiponectin in different tissue [4]. 

Adiponectin inhibits macrophage activation through the NF-*κ*B pathway [5] resulting in markedly decreased uptake of oxidized low-density lipoproteins and foam cell formation [6], reduces the production and activity of tumor necrosis factor *α* (TNF-*α*), and inhibits (IL-6) production [7]. It is known to exert antiapoptotic effects and to prevent myocardial apoptosis in response to myocardial ischemia reperfusion injury [8]. 

Plasma adiponectin levels are decreased in critically ill patients during the acute phase of their illness [9]. In contrast, adiponectin levels are elevated and positively correlated with inflammatory markers in chronic inflammatory diseases that are unrelated to increased adiposity [10]. 

In the current study, we sought to establish the pattern of changes in adiponectin levels in the context of the inflammatory response in pediatric patients with congenital heart defects undergoing OHS with CPB, focusing on the immediate postoperative period. We related the observed values to clinical outcome and to postoperative changes in other inflammatory markers.

## 2. Materials and Methods

### 2.1. Patients

24 consecutive children who underwent OHS with CPB for the correction of congenital heart defects were enrolled. Exclusion criteria included need for inotropic support or mechanical ventilation prior to surgery, recent cardiac arrest, sepsis, bout of severe hypotension or infection within one week of surgery, and weight of less than 2 kg. None of the patients received glucocorticoid therapy before enrollment. Preoperative informed consent was obtained from the parents of all children. The study was approved by the Chaim Sheba Medical Center ethics committee and the Israeli Ministry of Health.

### 2.2. Intraoperative Management

Induction and maintenance of anesthesia were carried out in a standard manner and consisted of weight-related dosages of fentanyl, midazolam, and pancuronium bromide. Routine operations were performed using a roller pump and membrane oxygenator. A single dose of cefazolin 0.5 mg/kg and methylprednisolone 30 mg/kg was administered. Five minutes prior to being connected to the Cobe Century 2000 CPB (Cobe Cardiovascular Inc, Arvada, CO, USA), patients were given 300 IU/kg bovine heparin in order to achieve an activated clotting time of 400–500 seconds. Surface body cooling was instituted with low ambient room temperature and a cooling mattress. The aorta was cross-clamped and a cold crystalloid antegrade cardioplegia solution (4°C) was infused through the aortic root in order to bring the body temperature to 25–27°C. Plegisol cardioplegia solution (Abbott Laboratories, North Chicago, IL, USA) was used for the first 17 CPBs, and Custodiol cardioplegia solution (Koehler Chemie, Alsbach-Haenlein, Germany) for the remaining 7. No modified ultrafiltration or leukocyte depletion techniques were employed for any of the patients during this study. 

### 2.3. Collection of Blood Samples

Serial blood samples were collected from arterial or venous lines 24 hours prior to surgery, after anesthesia induction, 1 and 2 hours while under CPB, upon arrival to the pediatric intensive care unit (PICU), and 6, 12, 24, and 96 hours afterwards. Blood was stored for 20 minutes at 4–8°C, centrifuged for 15 minutes at 2700 rpm, separated, and frozen at −70°C until use. 

Total serum adiponectin was measured using human adiponectin radioimmunoassay (Millipore, St. Charles, MO, USA) [11]. The sensitivity of the assay was 1 *μ*g/L, and the within and between assay variations were 6.9% and 9.3%, respectively. CRP levels were determined with a hsCRP kit (Olympus, AU2700 immunoanalyzer). The within and between assay variations were 1.10% and 1.83%, respectively. sP selectin, tissue-type plasminogen activator (tPA), monocyte chemoattractant protein-1 (MCP1), soluble CD40 (sCD0), and soluble vascular cell adhesion molecule 1 (sVCAM1) levels were measured using the Human Chemokines 6plex FlowCytomix Multiplex kit (Bender MedSystems Inc). 

The distribution of adiponectin multimers in serum samples was analyzed under nonreducing and nondenaturing conditions [11]. The relative proportion of each multimer to total adiponectin was calculated by dividing band densitometry of the multimer by total density in each lane. 

### 2.4. Selected Patient Data

Demographic data, medical history, and relevant laboratory parameters were recorded preoperatively. Each child's vital signs and laboratory results were monitored continuously while in the PICU. These included arterial blood gas, complete blood count, blood chemistry, duration of ventilation and stay at the PICU, rectal temperature, blood pressure, pulse, blood product infusion, use of intravenous solutions, and the inotropic score (IS) which was calculated as described by Wernovsky et al. [12]. 

### 2.5. Statistical Analysis

All parameters are expressed as mean values ± SD. CRP is expressed as median (25th and 75th percentiles). Analysis of variance (ANOVA) with repeated measures was applied for the testing of the time effect on adiponectin, log CRP, length of ventilation, difference between cardioplegia solutions, differences between cyanotic and acyanotic heart defects, IS, duration of operation, and length of stay at the PICU. Analysis of covariance (ANCOVA) with age as covariate was applied for the testing of time effect on adiponectin depending on the length of time on ventilation and length of stay in the PICU. Pearson correlations were used to test linear association between adiponectin and length of stay at the PICU, hours of ventilation, IS, weight, age, sVCAM1, and duration of surgery. A *P* value of less than 0.05 was considered significant. In addition, we separated our study group into two subgroups according to the median stay in the PICU in days (*N* = 5), hours ventilated (*N* = 22), and minutes on CPB (*N* = 72) and compared adiponectin levels between these two subgroups. 

## 3. Results

Twenty-four children, aged 1 month–11 years, met the study inclusion criteria. Patients' characteristics are summarized in Table 1. Individual patients' data is presented in Table 2. The surgical and clinical outcomes were favorable with the exception of one fatality.

### 3.1. Adiponectin Levels

**Figure 1:**
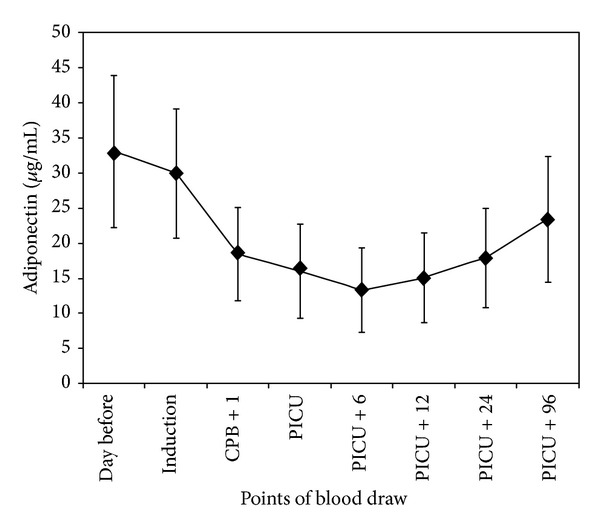
Time course of adiponectin serum concentrations in patients undergoing cardiopulmonary bypass (CPB). PICU = pediatric intensive care unit.

**Figure 2:**
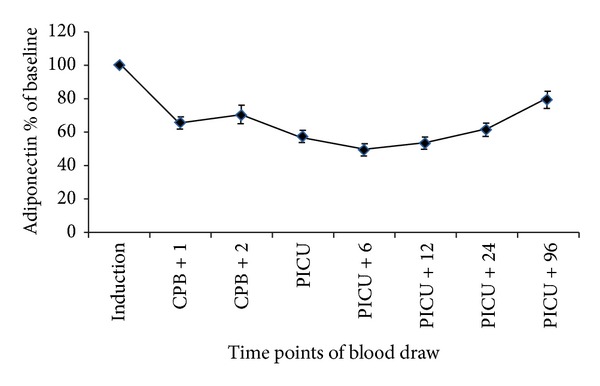
Time course of adiponectin serum concentrations as percentage of baseline in patients undergoing cardiopulmonary bypass (CPB). PICU: pediatric intensive care unit.

Table 2 and Figure 1 depict the changes in serum adiponectin levels that occurred over the study period. Adiponectin levels were highest before surgery, declining significantly (*P* = 0.01) from the time of induction of anesthesia up to 6 hours after the child's arrival to the PICU. Adiponectin levels at 96 hours after the arrival to the PICU were still significantly lower (*P* = 0.006) than those obtained at baseline. These observations held true when adiponectin levels were presented as percent of baseline (Figure 2).

### 3.2. Anthropometric Data

Baseline adiponectin negatively correlated with weight (*r* = −0.419, *P* = 0.042) and age (*r* = −0.545,  *P* = 0.006). There were no gender-based differences in adiponectin levels. 

### 3.3. Inflammatory Markers

CRP levels were evaluated at baseline (median 25th and 75th percentiles) (1.94 mg/L, 0.99, 3.39), upon arrival to the PICU (1.99 mg/L, 0.98, 2.91), and 24 and 96 hours after arrival to the PICU (33.3 mg/L, 22.91, 53.88, and 33.14 mg/L, 23.46, 62.43, resp.). Log transformation was performed in order to obtain normal distribution. At 24 hours after arrival to the PICU, log CRP and adiponectin demonstrated a trend of correlation (*r* = 0.41, *P* = 0.06); at this point adiponectin levels began to rise, while CRP levels were at their peak. 

**Figure 3:**
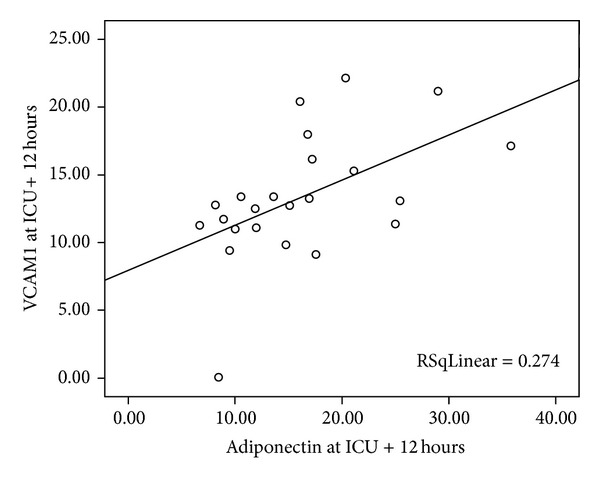
The correlation between adiponectin levels and sVCAM 1 levels 12 hours after arrival to the PICU.

Adiponectin levels at induction and sVCAM 1 levels at 12 and 24 hours after arrival at the PICU correlated positively (*P* = 0.01, *P* = 0.006) as did the adiponectin levels at 12 hours after arrival to the PICU and sVCAM 1 levels at 12 and 24 hours after arrival to the PICU (*P* = 0.017, *P* = 0.036) (Figure 3). Moreover, there was a positive correlation between sVCAM 1 at 12 hours after arrival to the PICU and adiponectin levels from the induction of anesthesia and up to 24 hours after arrival to the PICU (*P* = 0.013, *P* = 0.035, *P* = 0.044, *P* = 0.17, and *P* = 0.044, resp.). 

No correlations were noted between adiponectin levels and the following parameters: white blood cell count, platelets, lactate, troponin, length of cross-clamp time, brand of cardioplegia used (Plegisol versus Custodiol) or type of cardiac defect (cyanotic versus acyanotic), sP selectin, tPA, MCP1, and sCD40.

### 3.4. Severity of the Postoperative Course

**Figure 4:**
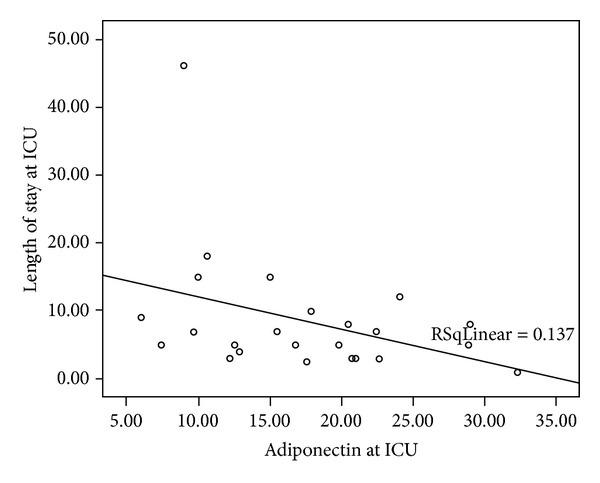
The correlation between adiponectin levels upon arrival to the PICU and length of stay in the PICU.

Using the well-established severity indicators, duration of CPB, duration of mechanical ventilation, length of stay at the PICU and IS [13], we found negative correlations between the duration of CPB and adiponectin levels at the end of surgery and at 6 hours after arrival to the PICU (*r* = −0.438, *P* = 0.037 and *r* = −0.488, *P* = 0.021, resp.). There was also a negative correlation between adiponectin levels and log of length of stay in the PICU, starting from the time of arrival to the PICU (*r* = −0.457,  *P* = 0.025) (Figure 4) and up to 12 hours afterwards (*r* = −0.495, *P* = 0.016). IS and adiponectin levels correlated negatively from the time of arrival to the PICU (*r* = −0.471, *P* = 0.02) and up to 24 hours afterwards, (*r* = −0.453, *P* = 0.03). No correlations were demonstrated between adiponectin levels and length of mechanical ventilation. Again, these observations remained unchanged when adiponectin was presented as percent of baseline levels (data not shown). Delta adiponectin levels (i.e., the difference between the levels 24 hours preoperation and at the time of arrival to the ICU) were positively correlated with length of CPB (*r* = 0.515, *P* = 0.007) and with the inotropic score (*r* = −0.394, *P* = 0.047), but not with the length of stay at the PICU.

Next, we divided the patients into two subgroups according to group median in each of the severity indicators and compared adiponectin levels between them. Adiponectin levels were significantly lower in the longer ventilation subgroup at arrival to the PICU and up to 24 hours afterwards (*P* = 0.029). No significant differences were noted after dividing the cohort based on median length of CPB and length of stay at the PICU. Finally, grouping the patients according to the use of inotropic agents revealed no significant difference between the patients who did not need inotropic assistance and those who did. 

Higher preoperative adiponectin levels were associated with a longer length of stay in the PICU (*P* < 0.001) and longer length of time on ventilation (*P* < 0.001). 

### 3.5. Molecular Forms of Adiponectin

**Figure 5:**
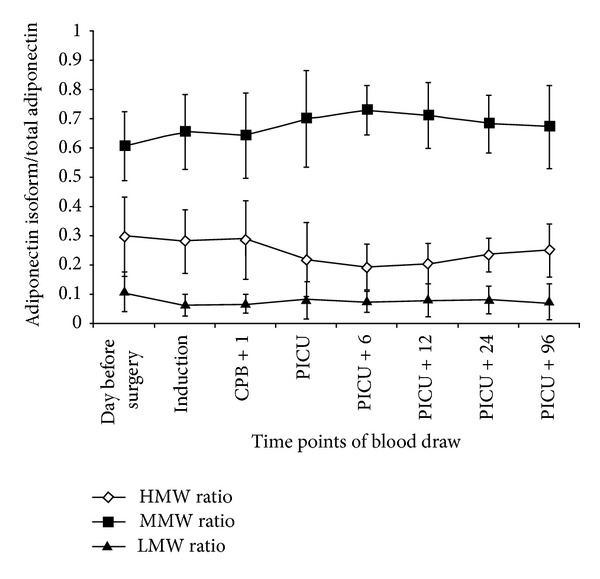
Time course of adiponectin isoforms/total adiponectin ratio in the study group.

Figure 5 depicts the changes in the molecular weight isoform ratio over time. We assessed the relative distribution of the three molecular isoforms of adiponectin at the 8 time points in 8 patients (4 boys and 4 girls) in order to exclude the possibility that an alteration in the concentration of one of adiponectin's isoforms was responsible for the change in total adiponectin levels. The relative distribution of the three isoforms was preserved at all time points and changes in the relative distribution of the three fractions were not significant over time.

## 4. Discussion

In the current study we longitudinally measured adiponectin levels and correlated them to clinical outcomes in pediatric patients undergoing OHS with CPB, a procedure which is expected to induce acute illness and inflammation [1]. Postoperative adiponectin levels were reduced over time compared to baseline levels. Similar results were recently observed by Caselli et al.; however, these authors measured adiponectin levels 3 days after surgery rather than during the immediate postoperative period as we did [14].

One possible explanation for the postoperative decline of adiponectin could be a reduction in adiponectin transcription due to the inflammatory process and the release of proinflammatory cytokines and endogenous cortisol. TNF-*α*, known to surge after CPB [15], suppresses the transcription of adiponectin in adipocytes [16]. In agreement with this hypothesis, Witasp et al. have recently demonstrated a significant decrease in adiponectin mRNA in adipose tissue during major abdominal surgery [17]. Alternatively, there may have been a decrease in adiponectin secretion from adipose tissue, or rapid degradation and clearance of adiponectin from the circulation due to the inflammatory process [18]. We established that changes in the secretion of the molecular forms of adiponectin were not responsible for the alterations in the total adiponectin levels, as we noted no change in the ratio of different adiponectin isoforms. 

Interestingly, adiponectin levels in our study began to rise faster than previously reported after the onset of a critical illness [9]; that is, levels had already started to increase 12 hours after the arrival to the PICU. 

Patients with a longer length of stay in the PICU and those with longer duration of ventilation had significantly higher preoperative levels of adiponectin and significantly lower adiponectin levels upon arrival to the PICU, even when age was taken into consideration. There is some evidence that patients who develop postoperative complications may be identified preoperatively based on the hypothesis that the primed immune system amplifies the immune response to cardiac surgical trauma. Bocsi et al. demonstrated elevated levels of the percentage of leukocytes that are neutrophils, the complement components C3 and C5 as well as TNF-*α* one day before surgery in patients that had a complicated postoperative course [19]. The amount of inflammatory markers released in critical care situations appears to be influenced genetically [20]. Thus, the host response to cardiac surgery is likely a result of interactions of the surgical procedure and innate patient characteristics which may have a genetic basis with carriage of specific functional polymorphic alleles in genes encoding for proteins involved in inflammation and vascular regulation, such as adiponectin.

Children with a more complicated course, as indicated by longer postoperative ventilation duration, had significantly lower postoperative adiponectin concentrations. Similarly, we observed negative correlations between adiponectin levels and clinical indicators of a more severe inflammatory process [19]—length of CPB, length of stay at the PICU, and IS. These findings support the notion that adiponectin participates in the inflammatory process that is set into motion after OHS and CPB. 

CRP, an acute phase reactant and a marker of the inflammatory process, has been shown to decrease immediately after OHS with CPB and to be elevated up to 5 days afterwards [21]. CRP levels negatively correlate with adiponectin levels in healthy men, obese women, and diabetic patients [22]. In the current study, the usual negative correlation between CRP and adiponectin was abolished following CPB. Adiponectin levels fell together with a slight drop in CRP levels and returned to near basal levels at the point when CRP was at its peak, demonstrating a trend for a positive correlation between the two 24 hours after arrival to the PICU. These results are in accordance with those found by Venkatesh et al. [20] and might indicate that adiponectin is a sensitive inflammatory marker for both the onset and the termination of the inflammatory process. The lack of the expected negative correlation between adiponectin and CRP could, however, be attributed to the time points of blood assessments that were chosen in this study. 

 We demonstrated a positive correlation between sVCAM-1 at 12 and 24 hours after arrival at the PICU and adiponectin levels up to 24 hours after arrival to the PICU. VCAM-1 is an endothelial ligand for integrins expressed on leukocytes and platelets, with the function of facilitation of endothelial adhesion of circulating leukocytes. The endothelial expression of VCAM-1 is increased in response to inflammatory cytokines, and the soluble ectodomain of VCAM-1 (sVCAM-1) is proteolytically shed from the cell surface into the circulation upon endothelial activation and injury. Elevated plasma sVCAM-1 level was reported in many diseases, including coronary and peripheral atherosclerosis, diabetes mellitus, systemic lupus erythematosus, and Crohn's disease, and it is now considered as a marker for endothelial injury under inflammatory processes [23]. There are contradictory reports regarding the association between adiponectin and sVCAM-1; adiponectin was found to suppress the adhesion of monocytes to endothelial cells by decreasing the TNF-*α*-mediated expression of sVCAM-1, thus reducing atherosclerosis [24]. On the other hand, Vaverkova et al. demonstrated a significant independent positive association of adiponectin with sVCAM-1 in patients at risk for coronary vascular disease [25]. We found no correlation between adiponectin and other inflammatory markers, such as white blood count, platelet count, sP selectin, MCP-1, sCD 40, or tPA.

Several limitations of this study should be considered. The reduction of adiponectin levels could be due to the pulse dose of methylprednisolone (30 mg/kg) which was administered in theater, in accordance with current practice [26]. The effects of glucocorticoids on the expression and secretion of adiponectin remain controversial. Dexamethasone has been shown to reduce adiponectin gene expression in murine adipocytes, and glucocorticoids were demonstrated to negatively regulate adiponectin mRNA in human adipose tissue [27]. A decrease of about 25% in adiponectin levels was noted by Fallo et al. when administering 25 mg of hydrocortisone to adults, lasting up to 120 minutes [27], while Uchida et al. demonstrated an increase in adiponectin after 3 days of steroid pulse therapy in patients with IgA nephropathy [28]. These differences could be related to adaptive mechanisms compensating for glucocorticoid-induced reduction in insulin sensitivity [29]. The fact that longer duration of stay at the PICU, longer duration of ventilation, and higher IS were correlated with lower adiponectin levels does not support the assumption that steroid therapy was solely responsible for the reduction in adiponectin levels, which lasted up to 96 hours after methylprednisolone administration, beyond the effect noted by Fallo et al. [27]. This fact further supports the role of inflammation in reducing adiponectin levels, rather than the effect of the single dose of methylprednisolone which was administered at the beginning of surgery.

## 5. Conclusions

We have demonstrated that a complicated postoperative course is associated with significantly lower postoperative adiponectin concentrations. Our data contributes to the accumulating evidence showing that adipose tissue is an active organ secreting multiple adipocytokines, which may have an impact on numerous pathways of response to critical illness. Overall, our findings raise the possibility that adiponectin may play a part in the response to CPB. The significance of this phenomenon, however, remains to be elucidated, and further efforts in basic research to account for the mechanisms beyond the clinical observation of low adiponectin levels in children following OHS are warranted.

## Figures and Tables

**Table 1:** Characteristics of the study patients.

	Mean ± SD	Range
Age (m)	20.08 ± 32.45	(1–132)
Sex (male/female)	12/12	
Weight (kg)	8.87 ± 6.75	(2.7–28)
*Type of surgery *		
Ventricular septal defect	3	
Atrial septal defect	1	
Tetralogy of Fallot	6	
Dextrotransposition of the great arteries	5	
Total anomalous pulmonary venous connection	2	
Pulmonic atresia	3	
Discrete subaortic stenosis	2	
Other	2	
CPB duration (min)	93.56 ± 52.43	(30–197)
Cross-clamp duration (min)	59.30 ± 48.24	(0–159)
PICU stay (days)	8.6 ± 9.08	(1–46)
Postoperative ventilation (hours)	92.02 ± 220.86	(0–1100)

CPB: cardiopulmonary bypass; PICU: pediatric intensive care unit.

**Table 2:** Adiponectin levels and weight *Z*-scores.

Patient number	Patient sex	Age (months)	Normative adiponectin levels	Baseline Adiponectin	Adiponectin at PICU	Adiponectin after 96 hours	Weight (Kg)	Weight *Z*-score
1	0	60	9.3	17.8	7.4	12.5	14	−2.07
2	0	1	11.2	32.7	10.6	24.8	3	−2.03
3	1	12	14.3	23.8	12.2	18.1	7.6	−2.85
4	0	37	11.2	27.5	15.5	16.6	11.7	−1.66
5	1	98	7.6	24.9	12.5	12.2	18.6	−2.73
6	1	132	7.8	16.9	12.9	15.9	28	−1.49
7	0	12	11.2	19.9	10	8	8.55	−1.01
8	1	1	26.7	26	9.7	17.5	2.7	−2.61
9	0	16	11.2	48.1	32.3	58	7.25	−3.83
10	1	1	26.7	44.1	17.9	35.9	3.4	−1.62
11	0	18	11.2	30	20.7	25	13.5	−1.87
12	1	12	14.3	31	16.8	24.2	7.58	−2.87
13	0	1	26.1	53.9	6	18.2	3.79	−0.69
14	0	18	11.2	31.5	17.6	20.9	10.1	−0.81
15	0	11	11.2	27.2	22.4	33.3	6.8	−2.82
16	1	2	26.7	43.3	28.9	26.3	4.2	−1.52
17	1	1	26.7	49.2	29	36.4	3.67	−1.22
18	1	10	14.3	46.1	20.5	28.6	8.8	−0.84
19	0	4	26.1	32.1	21	24.7	4.3	−2.61
20	0	3	26.1	38.8	15	27.4	3	−3.86
21	1	1	26.7	38.8	24.1	13.6	25	−2.88
22	1	3	26.7	32.2	9	21.4	5	−1.39
23	1	6	26.7	45.2	22.6	42.2	6.25	−1.98
24	0	9	11.2	24	19.8	No data	6.19	−2.82
